# Supplementary material for: BRAF Modulates Lipid Use and Accumulation
Source: Cancers (Basel). 2022 Apr 23;14(9):2110. doi: 10.3390/cancers14092110 (PMC9105200; doi:10.3390/cancers14092110)
Supplement: Supplementary file 1 [file cancers-14-02110-s001.zip › Supplemental Table S2.pdf]

Supplemental Table S2. Primer Sequences

Gene	Primer Type	Primer Sequence
ABHD12	Sense	5’ TGATATACCGATACTTCCCTG 3’
	Anti-Sense	5’ TGTATAGCTTTCTACCGAGATG 3’
ACACA	Sense	5’ CTGTATGAGAAAGGCTATGTG 3’
	Anti-Sense	5’ AACCTGTCTGAAGAGGTTAG 3’
ACACB	Sense	5’ GCATGAAGGACATGTATGAG 3’
	Anti-Sense	5’ AGGGATGTAGATGAGAATGG 3’
AIFM2	Sense	5’ GGAGTCGAGTTCTAAAACAAC 3’
	Anti-Sense	5’ TCTCTTCCACAGTTAACCAG 3’
ANGPTL3	Sense	5’CAAGGGTTTAATGTCTACTGTG 3’
	Anti-Sense	5’ CCCAACCAAAATTCTCCATC 3’
BSCL2	Sense	5’ CTGTTTCATGTTATACCAGAGG 3’
	Anti-Sense	5’ GTTGGCACATACGAATTCTC 3’
CD36	Sense	5’ CATTTGCAGGTCTATCTACG 3’
	Anti-Sense	5’ CAATGTCTAGCACACCATAAG 3’
CPT1a	Sense	5’ GGGAGGAATACATCTACCTG 3’
	Anti-Sense	5’ GAAGACGAATAGGTTTGAGTTC 3’
CPT1b	Sense	5’ ACTAACTATGTGAGTGACTGG 3’
	Anti-Sense	5’ TGGCATAATAGTTGCTGTTC 3’
DGAT1	Sense	5’ ACCTACCGAGATCTCTATTAC 3’
	Anti-Sense	5’ CATGGAGTTCTGGATAGTAGG 3’
DGAT2	Sense	5’ CTACTTCCGAGACTACTTTCC 3’
	Anti-Sense	5’ CTGTGCTGAAGTTACAGAAG 3’
FAS	Sense	5’ TAGATGCCTCAAATCTTAGC 3’
	Anti-Sense	5’ TTTTAGCTTCCTGGATTGTC 3’
FASL	Sense	5’ TGAAAAGCAAATAGCCAACC 3’
	Anti-Sense	5’ TATACTTCACTCCAGAGATCAG 3’
LPCAT3	Sense	5’ CAGAAGACTATGATAACCGC 3’
	Anti-Sense	5’ TTCATCGAAGCCATTAAAGC 3’
MBOAT7	Sense	5’ CACAGTACATCTACAAGAGC 3’
	Anti-Sense	5’ CATGAAGCTTAGGTAGTAACC 3’
NR1H2	Sense	5’ TCACCCACTATTAAGGAAGAG 3’
	Anti-Sense	5’ TCTAAGATGACCACGATGTAG 3’
NR1H3	Sense	5’ GATGTTTCTCCTGATTCTGC 3’
	Anti-Sense	5’ CTCCAACCCTATCCCTAAAG 3’
PLA2g2a	Sense	5’ CAAGAAACCATACCACCATC 3’
	Anti-Sense	5’ TCTTAAGCCGAATCATTTCC 3’
PLA2g4a	Sense	5’ CAAAATAGGATGAGCATGACC 3’
	Anti-Sense	5’ CTCATATGGACTAAATTCCACC 3’
PLA2g6	Sense	5’ GAACCAGGTAAACAACCAAG 3’
	Anti-Sense	5’ ATAATCATTTCAGCACACCC 3’
PLIN1	Sense	5’ CTATGAGAAGGGTGTACAGG 3’
	Anti-Sense	5’ CTTTTCTGGAGGGTATTGAAG 3’
PPARγ	Sense	5’ AAAGACAACGGACAAATCAC 3’
	Anti-Sense	5’ GGGATATTTTTGGCATACTCTG 3’
RXRA	Sense	5’ TAACAGAGCTGGTGTCTAAG 3’
	Anti-Sense	5’ TTAGAGTCAGGGTTGAACAG 3’

RXRB	Sense	5’ AATAAAGGGGTAGTGAAGGG 3’
	Anti-Sense	5’ CCCTGACATTTAAGTCAACC 3’
SCD1	Sense	5’ AGAATATCCTGGTTTCCCTG 3’
	Anti-Sense	5’ CTAAGACAGTAGCCTTAGAAAC 3’
SHH	Sense	5’ GGGAAGATCACAAGAAACTC 3’
	Anti-Sense	5’ TTAACTTGTCTTTGCACCTC 3’
SLC27A1	Sense	5’ ATGTGCTCTATGACTGCC 3’
	Anti-Sense	5’ TATGTACTGCACTACCGTG 3’
SLC27A5	Sense	5’ ACCTCTGTACCATACGATAG 3’
	Anti-Sense	5’ CCACATACAAGATCACTGTTAC 3’
SQSTM1	Sense	5’ AATGTGATCTGTGATGGTTG 3’
	Anti-Sense	5’ GAGAGAAGCTATCAGAGAGG 3’
SREBF1	Sense	5’ AATAAATCTGCTGTCTTGCG 3’
	Anti-Sense	5’ CCTTCAGTGATTTGCTTTTG 3’
SREBF2	Sense	5’ TGGTAAATGGTGTGATTGTC 3’
	Anti-Sense	5’ GATAAGCAGGTTTGTAGGTTG 3’
TLR2	Sense	5’ CTAGAAGTGGAAAAGATGTCG 3’
	Anti-Sense	5’ TAGCATCCTCTGAGATTTGAC 3’
