# Supplementary figures and images for: BRAF Modulates Lipid Use and Accumulation
Source: Cancers (Basel). 2022 Apr 23;14(9):2110. doi: 10.3390/cancers14092110 (PMC9105200; doi:10.3390/cancers14092110)

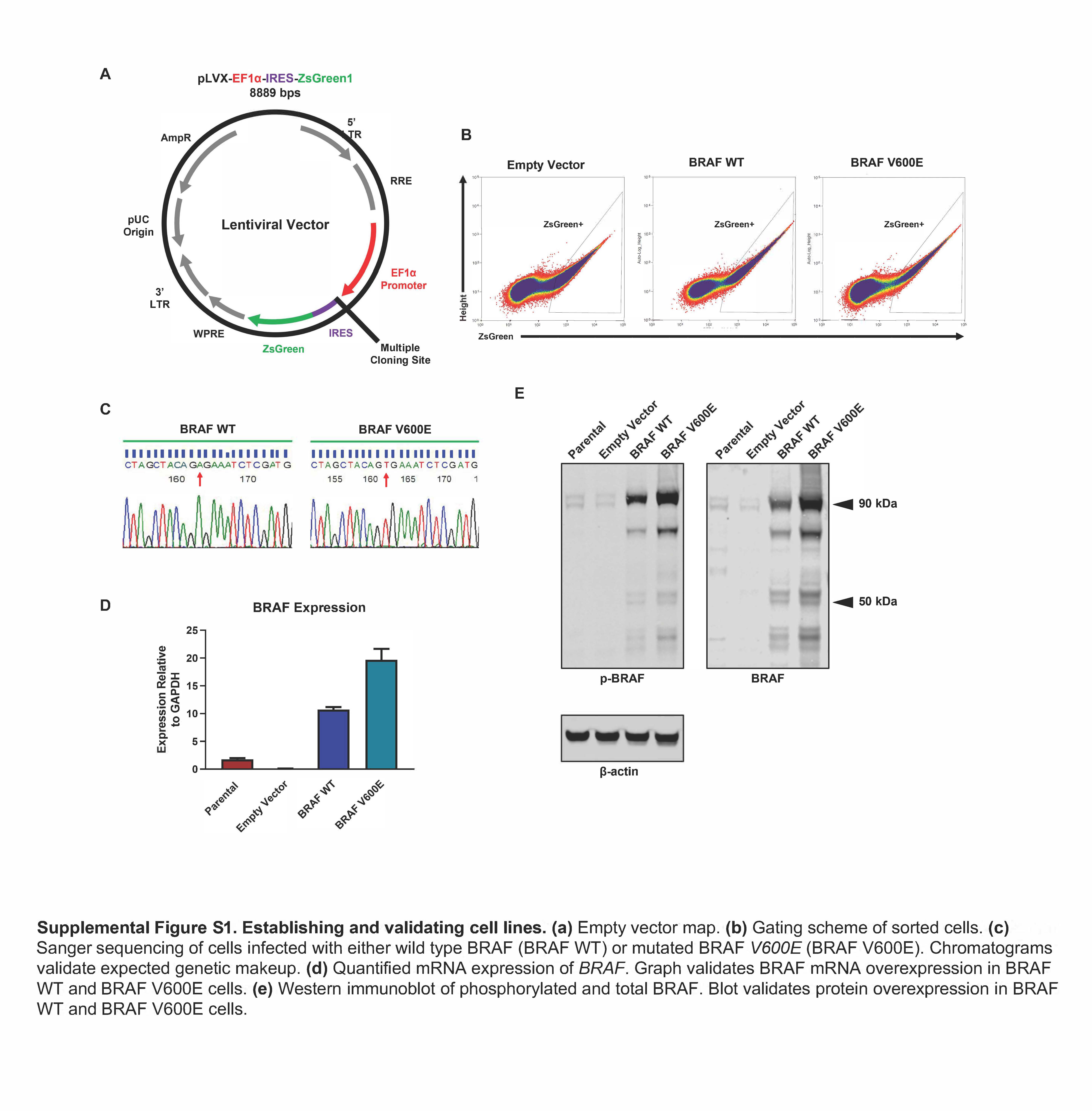

Supplement: Supplementary file 1 [file cancers-14-02110-s001.zip › Figure S1.jpg]

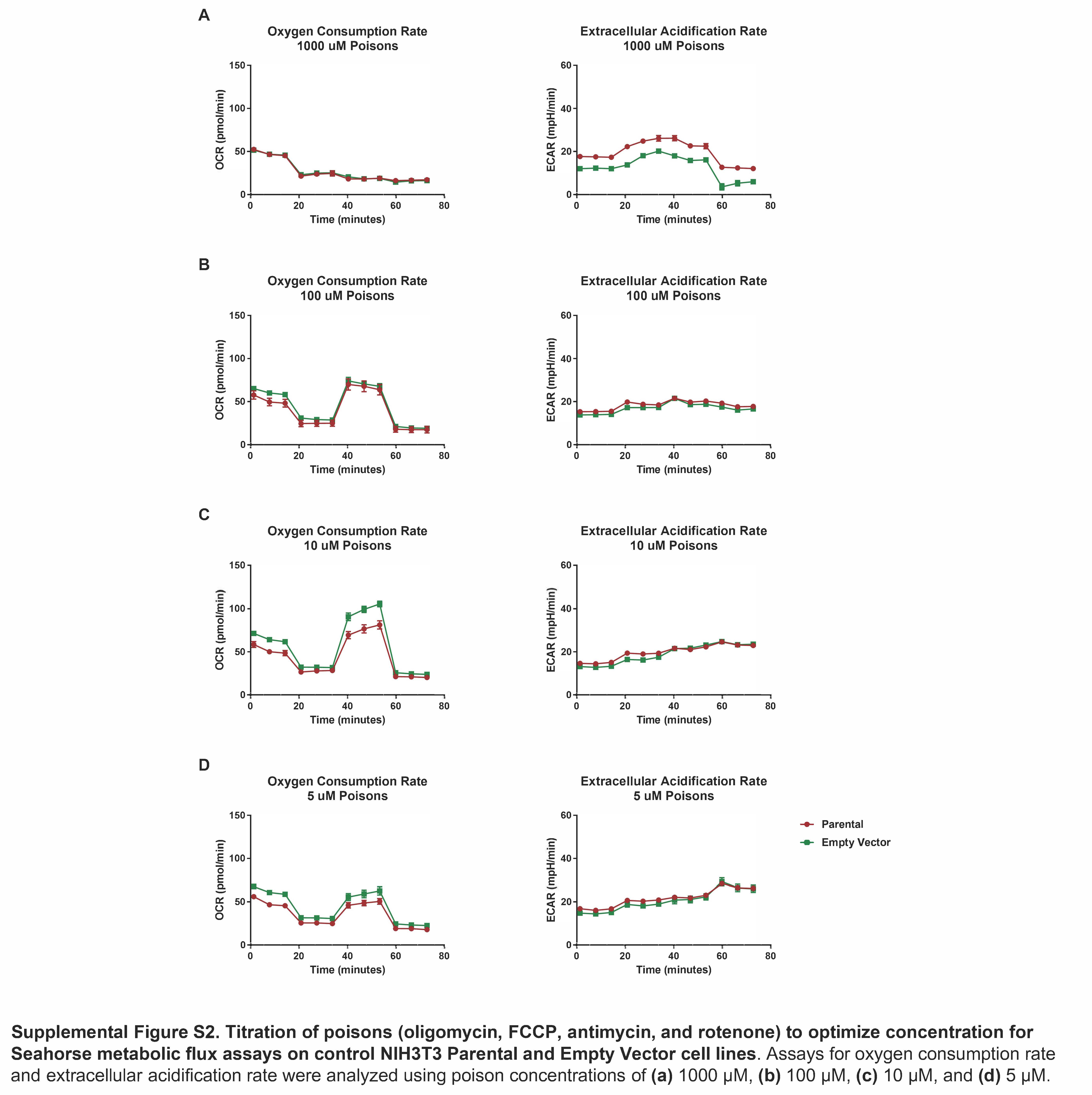

Supplement: Supplementary file 1 [file cancers-14-02110-s001.zip › Figure S2.jpg]

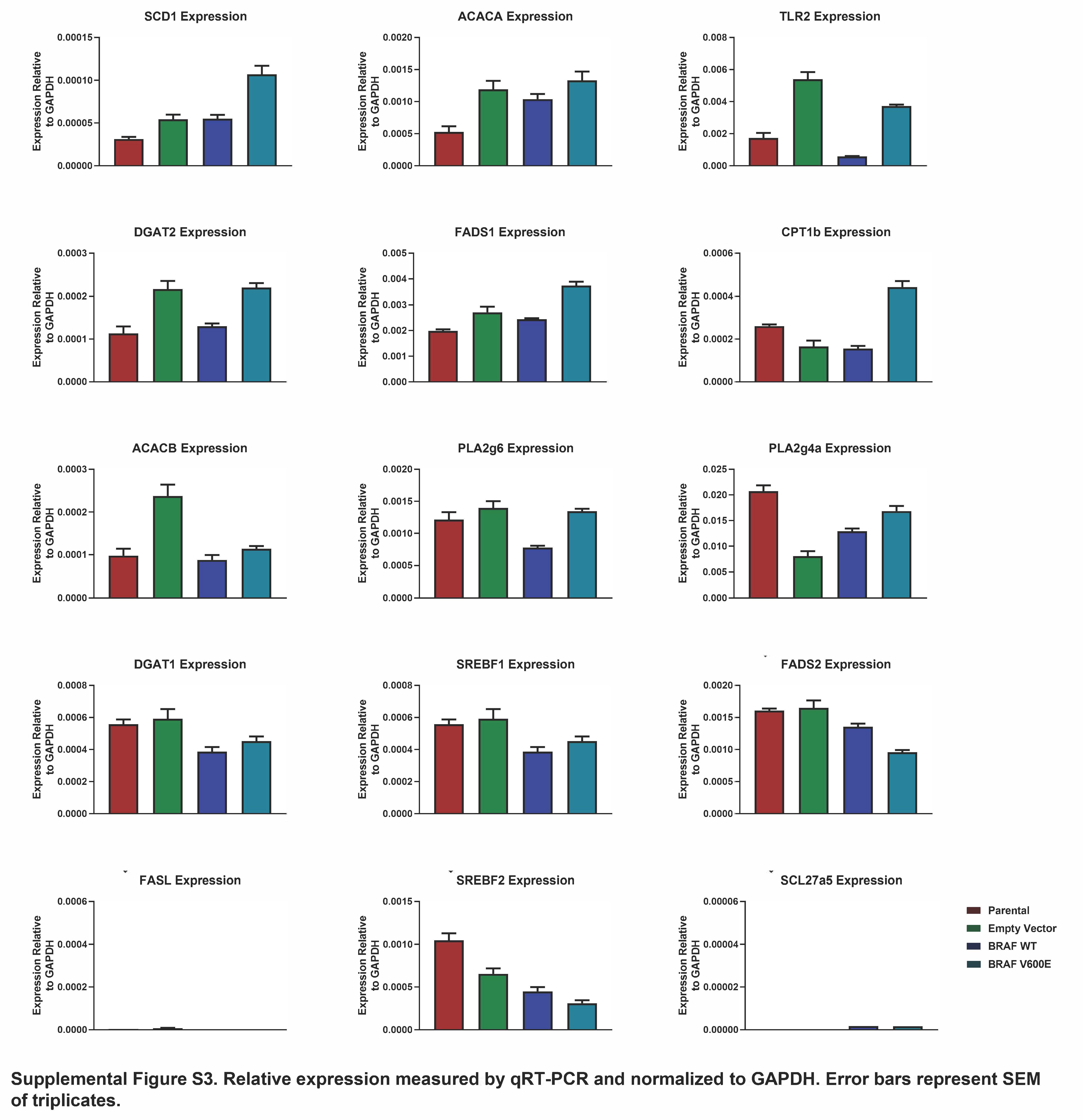

Supplement: Supplementary file 1 [file cancers-14-02110-s001.zip › Figure S3.jpg]

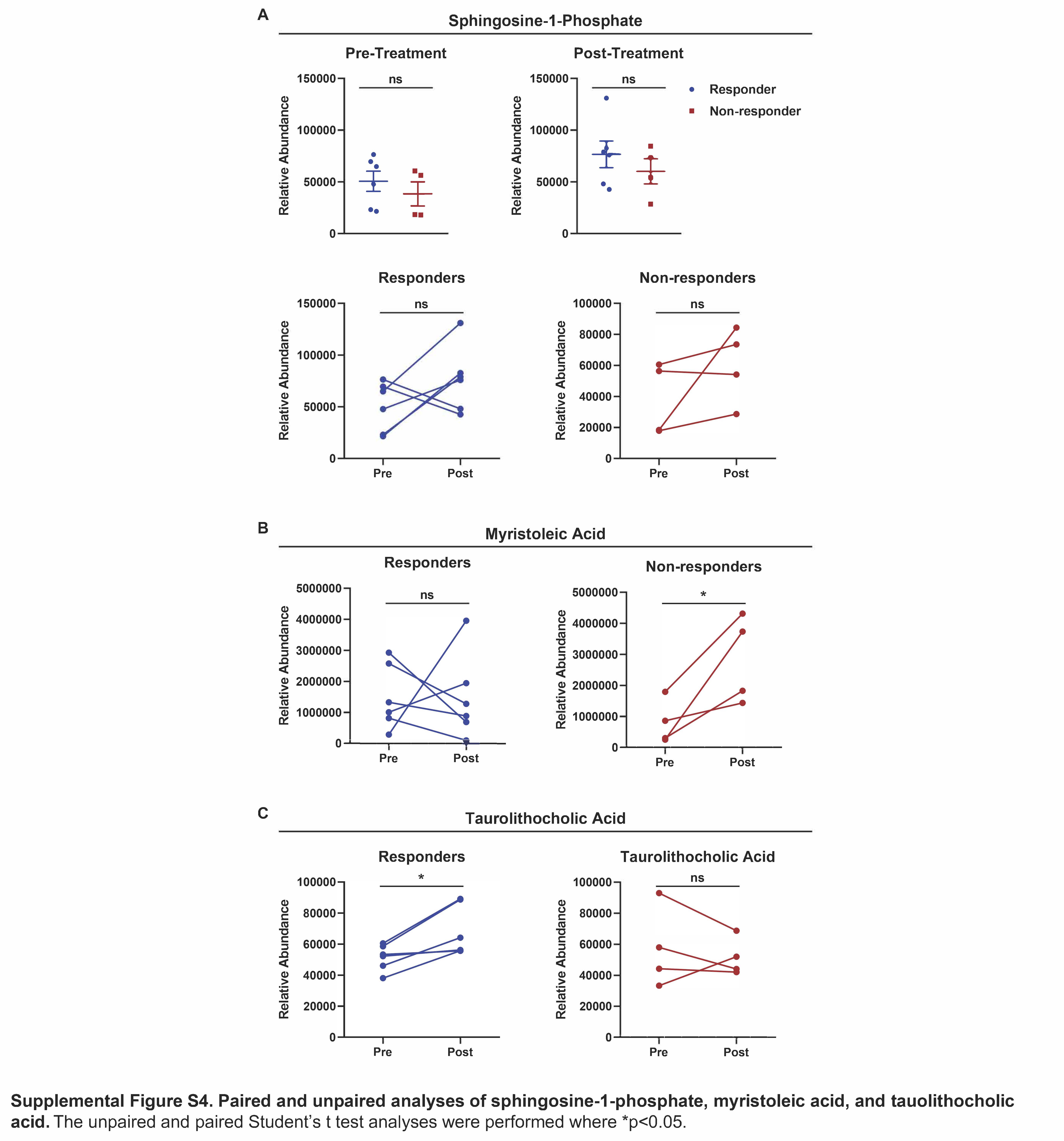

Supplement: Supplementary file 1 [file cancers-14-02110-s001.zip › Figure S4.jpg]

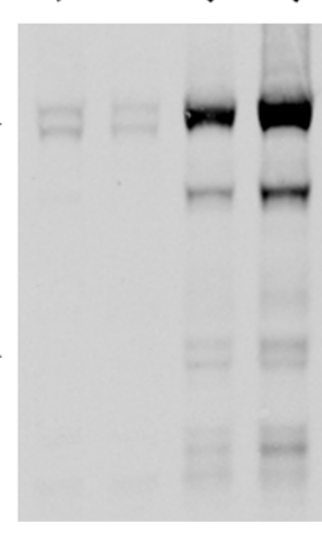

Supplement: Supplementary file 1 [file cancers-14-02110-s001.zip › Original blots of Figure S1/uncropped pBRAF.png]

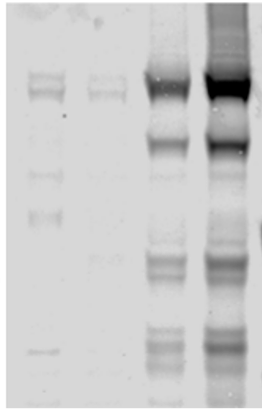

Supplement: Supplementary file 1 [file cancers-14-02110-s001.zip › Original blots of Figure S1/uncropped tBRAF.png]
